# Alterations in Auxin Homeostasis Suppress Defects in Cell Wall Function

**DOI:** 10.1371/journal.pone.0098193

**Published:** 2014-05-23

**Authors:** Blaire J. Steinwand, Shouling Xu, Joanna K. Polko, Stephanie M. Doctor, Mike Westafer, Joseph J. Kieber

**Affiliations:** Biology Department, University of North Carolina, Chapel Hill, North Carolina, United States of America; University of Nottingham, United Kingdom

## Abstract

The plant cell wall is a highly dynamic structure that changes in response to both environmental and developmental cues. It plays important roles throughout plant growth and development in determining the orientation and extent of cell expansion, providing structural support and acting as a barrier to pathogens. Despite the importance of the cell wall, the signaling pathways regulating its function are not well understood. Two partially redundant leucine-rich-repeat receptor-like kinases (LRR-RLKs), FEI1 and FEI2, regulate cell wall function in *Arabidopsis thaliana* roots; disruption of the FEIs results in short, swollen roots as a result of decreased cellulose synthesis. We screened for suppressors of this swollen root phenotype and identified two mutations in the putative mitochondrial pyruvate dehydrogenase E1α homolog, *IAA-Alanine Resistant 4 (IAR4)*. Mutations in *IAR4* were shown previously to disrupt auxin homeostasis and lead to reduced auxin function. We show that mutations in *IAR4* suppress a subset of the *fei1 fei2* phenotypes. Consistent with the hypothesis that the suppression of *fei1 fei2* by *iar4* is the result of reduced auxin function, disruption of the *WEI8* and *TAR2* genes, which decreases auxin biosynthesis, also suppresses *fei1 fei2*. In addition, *iar4* suppresses the root swelling and accumulation of ectopic lignin phenotypes of other cell wall mutants, including *procuste* and *cobra*. Further, *iar4* mutants display decreased sensitivity to the cellulose biosynthesis inhibitor isoxaben. These results establish a role for *IAR4* in the regulation of cell wall function and provide evidence of crosstalk between the cell wall and auxin during cell expansion in the root.

## Introduction

Cell expansion plays a critical role in plant growth and development. The direction and extent to which cells expand is controlled by the rigid, yet highly dynamic cell wall. The cell wall is a major determinant of cell size and shape and consequently overall plant morphology. In roots, the architecture of the cell wall permits longitudinal cell elongation while restricting radial expansion, which leads to highly asymmetric, anisotropic growth [1]–[4].

Plant cell walls are composed primarily of load-bearing cellulose microfibrils, cross-linking hemicelluloses, and pectins. Together with a relatively small number of structural proteins, this matrix of polysaccharides lends the wall the strength and rigidity that is required for structural support and plant defense, while simultaneously allowing cells to expand as plants grow and develop [5]. During cell expansion, wall polymers are actively remodeled and rearranged and their synthesis is altered in response to both developmental and environmental cues [6]. The ability of cell walls to maintain structural integrity and function properly as changes in the architecture of the cell wall occur suggests that there is a sensing and feedback system in place to perceive and respond to changes in the wall. Despite a crucial role in the maintenance of plant cell wall function, our current understanding of the components and mechanisms involved in the perception of and response to regulatory input from the wall remains poorly understood.

Several members of the receptor-like kinase (RLK) family have been implicated as sensors of signals from the cell wall. In Arabidopsis, the RLK family is comprised of approximately 600 members, many of which have been shown to act in a variety of different signaling pathways that function throughout plant development [7]. Of those, members of three different sub-families have been implicated in regulating cell wall function. The wall-associated kinases (WAKs) are tightly bound to the cell wall and are required for normal cell expansion [8]–[10]. In addition to the WAKs, four members of the *Catharanthus roseus* RLK1-Like (*Cr*RLK1L) subfamily (*HERCULES1*, *HERCULES2*, *FERONIA*, and *THESEUS1*) and two members of the leucine-rich repeat (LRR) subfamily (*FEI1* and *FEI2*) have been implicated in cell wall signaling. Although members from each of the three RLK subfamilies are required for cell expansion, only *THESEUS1 (THE1)*, its close homologs, and the FEIs have been linked to cell wall synthesis [11]–[13].

Mutations in *THE1* suppress ectopic lignin deposition and restore hypocotyl elongation in cellulose-deficient mutants, but do not restore cellulose biosynthesis in the *procuste* mutant [11]. These data suggest that *THE1* plays a role in sensing and actively responding to changes in the cell wall. Disruption of both *FEI1* and *FEI2* leads to a loss of anisotropic growth in rapidly expanding cells of the root elongation zone, but also affects cell expansion in the stamen filament and the hypocotyl of dark-grown seedlings [12]. In addition, the roots of double *fei1 fei2* mutants display ectopic lignin deposition, are hypersensitive to the cellulose synthesis inhibitor isoxaben, and synthesize less cellulose as compared to wild-type roots when seedlings are grown under non-permissive conditions of elevated salt or sucrose [12]. Further, disruption of *FEI2* leads to a reduction in the rays of cellulose observed in the mucilage of wild-type seeds [14], [15]. These data suggest that FEI1 and FEI2 positively regulate cell wall function by promoting cellulose synthesis.

The fasciclin-like GPI-anchored extracellular protein SOS5 acts in the FEI pathway to regulate cell wall synthesis [12]. Like *fei1 fei2, sos5* mutants display short, swollen roots when grown under the restrictive conditions of elevated salt or sucrose, and this phenotype is reversed in both mutants by blocking ethylene biosynthesis, but not ethylene perception. SOS5 also regulates the synthesis of cellulose during the production of seed coat mucilage [14]. Introduction of *sos5* into the *fei1 fei2* mutant does not cause an additive phenotype, in contrast to other mutants affecting cellulose biosynthesis such as *cobra* [12]. The non-additive phenotype of *fei1 fei2* and *sos5* mutations suggests that the FEI RLKs act in a linear pathway with SOS5 to regulate cellulose synthesis. Taken together, these data suggest an important role for the FEI RLKs/SOS5 pathway in positively regulating cellulose synthesis.

In order to better understand the FEI signaling pathway, we sought to uncover additional components involved in regulating cell wall synthesis in the root. Here we describe the identification and characterization of a suppressor of the *fei1 fei2* mutant. We show that mutations in the previously characterized *IAA-Alanine Resistant 4* (*IAR4*) gene, encoding a putative mitochondrial E1α pyruvate dehydrogenase subunit, suppress the defects in root anisotropic cell expansion exhibited by *fei1 fei2*. *IAR4* was originally identified in a genetic screen for IAA conjugate-resistant mutants [16] and was subsequently identified as an enhancer of *tir1* auxin resistance [17]. Although the precise role of *IAR4* in the auxin biosynthesis pathway remains unclear, *iar4* mutants display phenotypes consistent with reduced endogenous auxin, accumulate IAA-amino acid conjugates, and are rescued by increasing endogenous IAA levels in the plant [16], [17]. Thus, IAR4 is predicted to play an important role in maintaining auxin homeostasis. Here we show that reduced auxin function, via either an *iar4* single or a *wei8/tar2* double mutant, suppresses growth isotropy of cell wall mutants, including *fei1 fei2*. Our results shed light on the role of auxin in regulating cell wall function in the Arabidopsis root.

## Results

### Isolation and characterization of *shou2*

**Figure 1:**
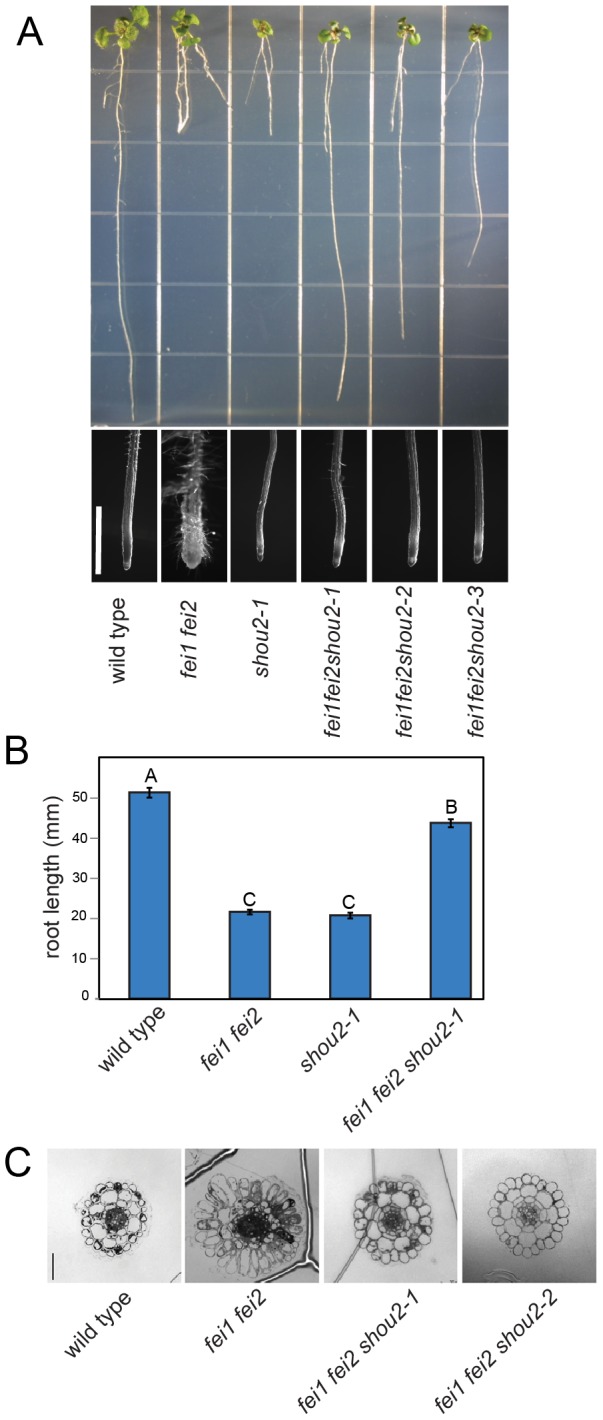
Isolation of the *shou2* suppressor. (**A**) Top: Phenotypes of indicated seedlings grown on MS medium containing 4.5% sucrose for three weeks. The bottom panels show a close-up of the root tips. Scale bar = 1 mm. (**B**) Quantification of total root length from (A). Values are the means (n = 150) ± SE. Different letters indicate significant differences between groups. Data were analyzed with one-way ANOVA and Tukey's post-hoc comparisons; *P*<0.05. (**C**) Transverse sections through the root elongation zone of wild type, *fei1 fei2, fei1 fei2 shou2-1*, and *fei1 fei2 shou2-2*. Scale bar = 50 µm.

In order to identify additional elements regulating cell wall function, we screened for suppressors of the swollen root phenotype of *fei1 fei2* mutants. An M_2_ population of ethyl methanesulfonate mutagenized *fei1 fei2* was screened for suppressors of the conditional short, swollen root phenotype of *fei1 fei2* seedlings. Eight independent suppressor lines that retested as robust *fei2 fei2* suppressors were identified from screening approximately 200,000 M_2_ seedlings representing 30,000 M_1_ seeds. We designated these suppressors *shou* mutations (the Chinese word for thin). These suppressors represented seven distinct loci, two of which were allelic and were designated *shou2-1 and shou2-2*. The *fei1 fei2 shou2-1* and *fei1 fei2 shou2-2* lines both had significantly fewer and shorter root hairs. The F_1_ of a backcross to the parental *fei1 fei2* line displayed a non-suppressed phenotype, and the suppressor phenotype segregated consistent with 3 non-suppressed: 1 suppressed ratio in the F_2_ progeny of this backcross, consistent with *shou2* acting as a single locus, recessive mutation. In addition to the suppression of root length (Fig. 1A, B), the *shou2* mutations also suppress the radial swelling (Fig. 1A) and the radial expansion of cells in the elongation zone (Fig. 1C) in *fei1 fei2* roots. We isolated the *shou2-1* mutation by backcrossing to the wild type. This *shou2-1* single mutant line displayed fewer and shorter root hairs, similar to the *fei1 fei2 shou2-1*. Intriguingly, under the non-permissive conditions used to assess the *fei* phenotype (grown on MS+4.5% sucrose), both the *fei1 fei2* and *shou2-1* parental seedlings displayed roots that were significantly shorter that their wild-type counterparts, despite the fact that the *fei1 fei2 shou2-1* triple mutants displayed nearly wild-type root elongation in the growth conditions used for the suppressor screen.

### *SHOU2* is allelic to *IAR4*

**Figure 2:**
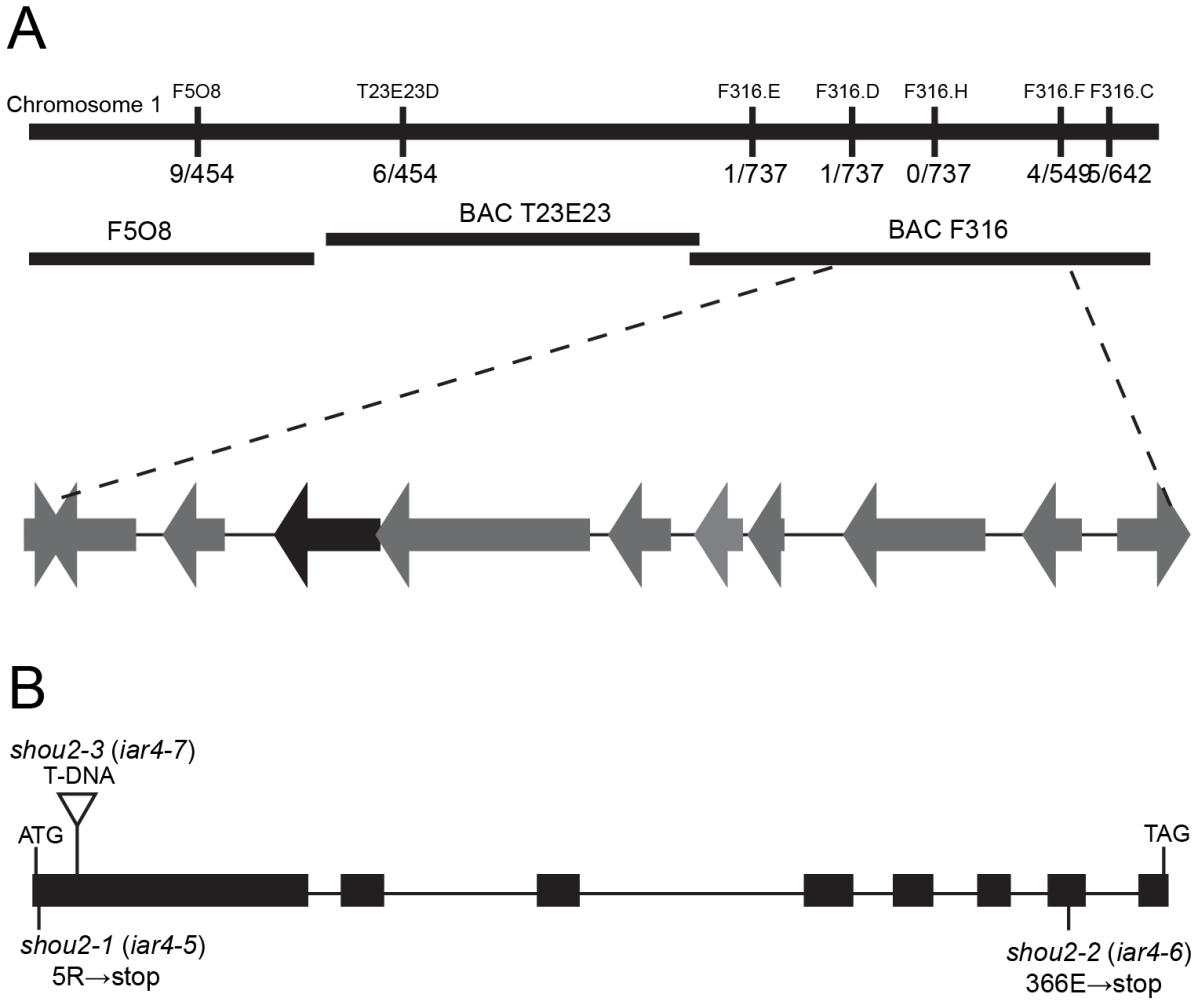
Positional cloning of *SHOU2*. (**A**) *shou2* was mapped to a region on chromosome 1 between markers F3I6.D and F3I6.F as described in the methods. The name of each DNA marker is shown above and the number of recombinants is indicated below the line. Open reading frames located between markers F3I6.D and F3I6.F are shown below BAC F316. (**B**) Structure of *SHOU2* gene. Boxes represent exons and lines represent the introns. The positions and changes of the three *shou* alleles are indicated. The triangle indicates the position of the DNA insertion in *shou2-3*. The corresponding allele numbers for the *iar4* designations are shown in parentheses.

We used a map-based positional cloning approach to isolate the *SHOU2* gene. The *fei1* and *fei2* mutations (isolated in the Columbia (Col) ecotype) were introgressed six times into the *Landsberg erecta* (L*er*) ecotype to generate a *fei1 fei2* plant that was largely L*er* except for small regions of DNA near the *fei1* and *fei2* mutations (see Materials and Methods). This line was crossed to *fei1 fei2 shou2* to generate a mapping population for *shou2-1*. Mapping with Col/L*er* SSLPs indicated that *SHOU2* was linked to the top of chromosome 1. Analysis of 350 *fei1 fei2* F_2_ progeny with additional molecular markers further delimited *SHOU2* to a 47 kb interval between the F3I6.D and F3I6.F markers (Fig. 2A; Table S1 in File S1). Sequencing of candidate genes within this region identified missense mutations in the first and seventh exon of *IAR4* (AT1G24180) in *fei1 fei2 shou2-1* and *fei1 fei2 shou2-2* respectively. The *shou2-1* allele contains a C→T transition in the fifth codon of the coding region of *IAR4*, which converts an arginine residue to a stop codon. The *shou2-2* mutation is the result of a G→A transition that is predicted to change a glutamate at position 366 to a stop codon (Fig. 2B). To confirm that *shou2* mutations correspond to AT1G24180, we examined the ability of an independent T-DNA insertional allele that contains a T-DNA insertion in the first exon of *IAR4* (SALK_091909) to suppress *fei1 fei2*. This *shou2-3* allele, which has no full-length *IAR4* transcript (Fig. S1 in File S1), was introduced into a *fei1 fei2* mutant line by crossing and the phenotype of the roots was examined in non-permissive conditions (Fig. 2B). Similar to the other alleles, *shou2-3* suppressed the root swelling phenotype of *fei1 fei2*, which confirms that mutations in *IAR4* correspond to *shou2*. Additionally, we performed crosses between *fei1 fei2 shou2-1*, *fei1 fei2 shou2-2 and fei1 fei2 shou2-3*. The F_1_ progeny of all three of these crosses displayed a wild-type root phenotype with no visible swelling of the tip when grown on 4.5% sucrose (Fig. S2 in File S1), which suggests that *shou2-1*, *shou2-2*, *shou2-3* are indeed allelic. To avoid confusion and to be consistent with prior studies, we re-named *shou2-1, shou2-2*, and *shou2-3*, to *iar4-5, iar4-6*, and *iar4-7* respectively.

### A role for auxin in regulating cell wall function

**Figure 3:**
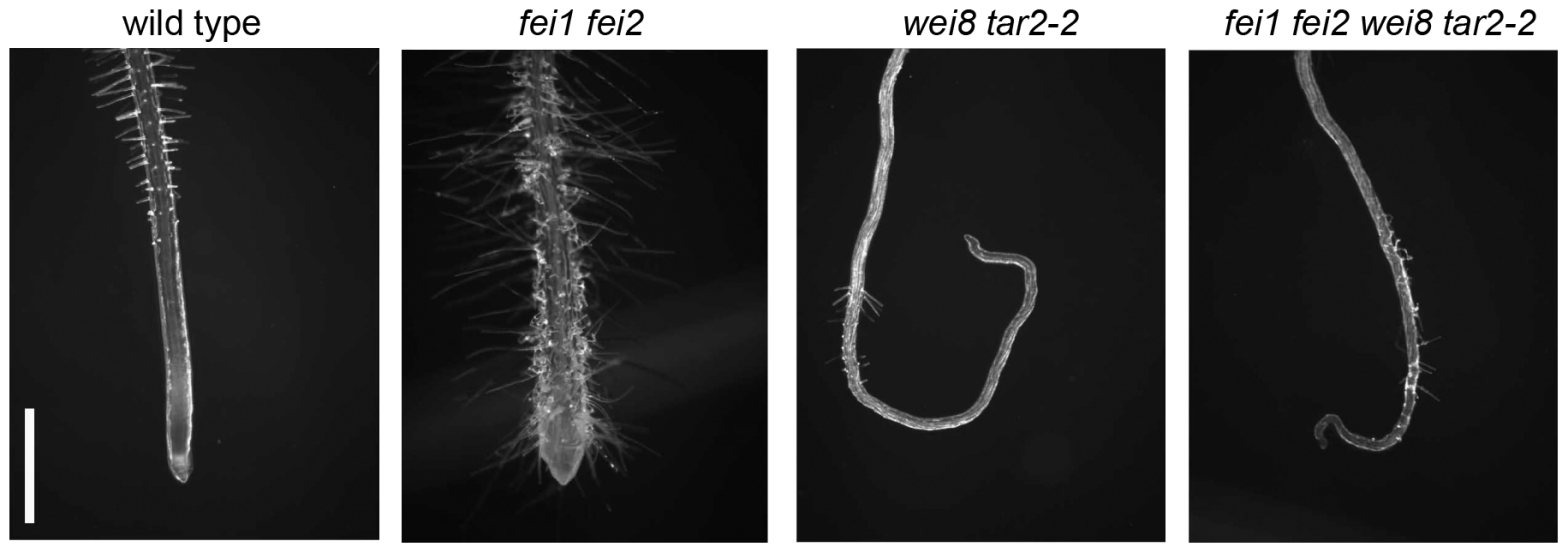
Mutations in the auxin biosynthetic genes *WEI8* and *TAR2* suppress *fei1 fei2*. Phenotypes of the root tips of the indicated seedlings four days after transfer from MS medium containing 0% sucrose to MS medium containing 4.5% sucrose. Scale bar = 1 mm.

As *IAR4* is involved in the maintenance of auxin homeostasis and mutations in *IAR4* restore anisotropic root growth in *fei1 fei2*, we hypothesized that a reduction in the level of endogenous IAA would also suppress the loss of growth anisotropy in *fei1 fei2*. To test this hypothesis, we examined the effect of mutations in the auxin biosynthetic genes *WEI8* and *TAR2* on the *fei1 fei2* root swelling phenotype. *WEI8* and *TAR2* are partially redundant genes that encode two of the five tryptophan aminotransferases (TAA1) essential for the major auxin biosynthesis pathway in plants. The level of IAA in the roots of double *wei8 tar2* mutants is reduced by 50% relative to the wild type [18], suggesting *WEI8* and *TAR2* are involved in auxin biosynthesis in roots. We generated a *wei8 tar2 fei1 fei2* quadruple mutant; when grown under restrictive conditions, the swelling of the root tip was suppressed in this quadruple *wei8 tar2 fei1 fei2* mutant (Fig. 3). The suppression of the *fei1 fei2* phenotype by *wei8* and *tar2* is similar to the suppression of *fei1 fei2* by iar4, which suggests that auxin is required for the radial cell expansion that occurs in response to decreases in cellulose synthesis in the absence of the FEI proteins. Consistent with this notion, growth of *fei1 fei2 iar4-5* seedlings at higher temperature (28°C), which has been shown to elevate endogenous auxin levels [19] and to suppress *iar4* phenotypes [17], blocks the suppression of root swelling the *iar4-5* mutant (Fig. S3A in File S1). Furthermore, the *fei1 fei2* mutant is slightly hypersensitive to auxin in a root elongation assay (Fig. S3B and C in File S1).

**Figure 4:**
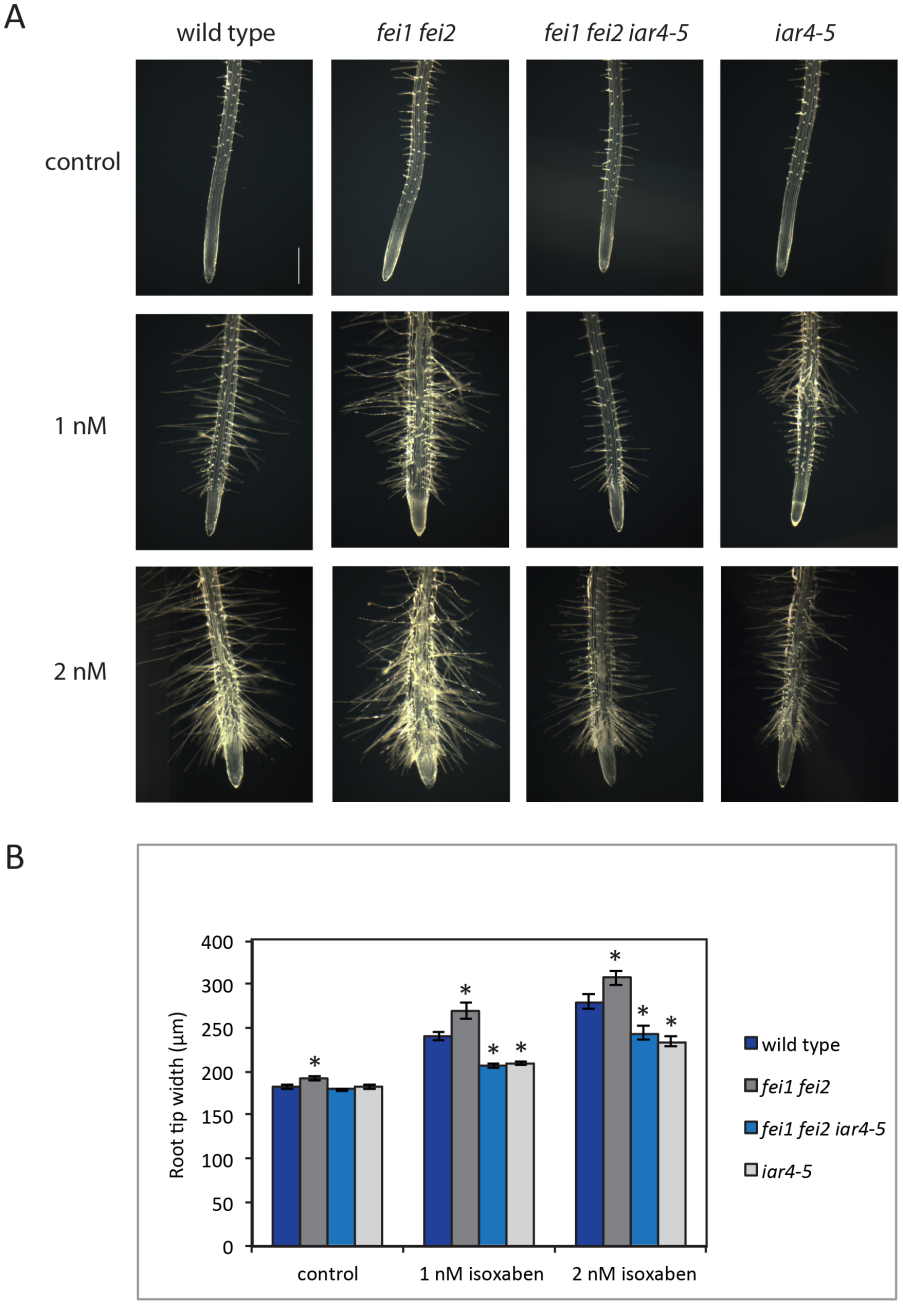
Mutations in *IAR4* confer resistance to isoxaben. (A) Root phenotypes of indicated seedlings germinated and grown for five days on MS medium with 0% sucrose and then transferred for 48 hours to MS medium with 0% sucrose containing either 0 nM (DMSO), 1 nM or 2 nM isoxaben. Representative root tips were imaged using dark field microscopy. Scale bar = 0.5 mm. (B) Quantification of the root tip swelling from (A). The width of the roots was measured at the level of the youngest root hair using ImageJ software [48]. Values are the means (n>8) ± SE. Data were analyzed by unpaired Student's t-test. Asterisks indicate significant differences relative to the wild type; *P*<0.05.

To further explore this hypothesis, we investigated the sensitivity of the *iar4-5* mutant to the cellulose synthesis inhibitor isoxaben. Previous work has shown that loss of growth anisotropy is exacerbated in cell wall mutants treated with isoxaben [20], [21]. Consistent with these results, *fei1 fei2* is hypersensitive to isoxaben [12]. However, in contrast to *fei1 fei2*, both the triple *fei1 fei2 iar4-5* and single *iar4-5* are partially resistant to the effects of isoxaben on root swelling (Fig. 4). The suppression of aberrant cell expansion by *iar4-5* suggests that the effect of the loss of cell wall integrity on root morphogenesis can be attenuated by a reduction in auxin function.

### The effect of *iar4* on other *fei1 fei2* phenotypes

**Figure 5:**
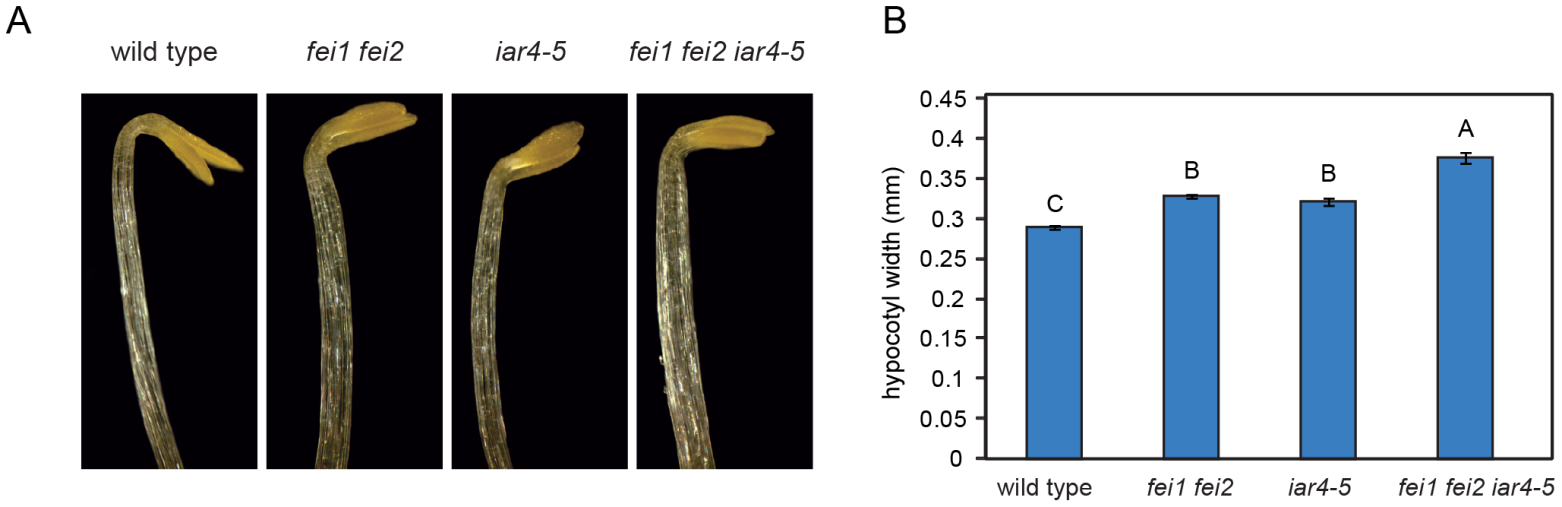
Effect of *iar4-5* mutation on the hypocotyl phenotype of *fei1 fei2*. (**A**) Seedlings of the indicated genotypes were grown for four days in the dark on MS medium with 0% sucrose. Representative seedlings were imaged using dark field microscopy. (**B**) Quantification of hypocotyl widths from seedlings grown as in (A). Values represent the mean (n = 20) ± SE. Different letters indicate significant differences between groups. Data were analyzed with one-way ANOVA and Tukey's post-hoc comparisons; *P*<0.05.

**Figure 6:**
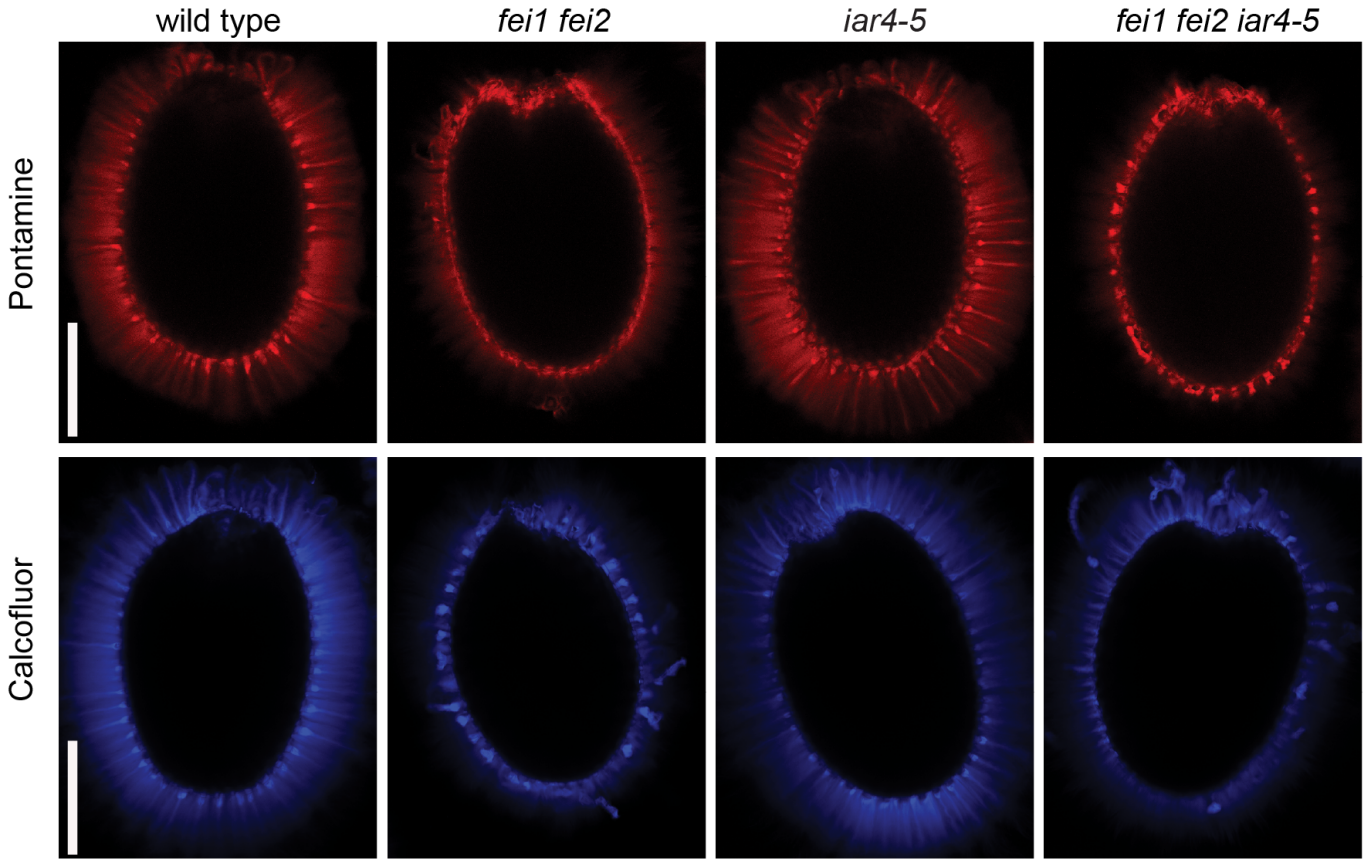
*iar4* does not suppress defects in the seed coat mucilage of *fei1 fei2*. Seeds of the indicated genotype were stained as described in the Methods with either Pontamine fast scarlet S4B or calcofluor as indicated and visualized by confocal microscopy. Scale bar = 0.2 mm.

We have previously shown that the FEI RLKs are required for proper hypocotyl cell expansion in etiolated seedlings and in anchoring pectin in seed coat mucilage to the seed surface [12], [14]. The hypocotyls of dark-grown *fei1 fei2* seedlings are significantly wider than those of the wild type [12]. In addition, mutations in *FEI2* lead to disruption of seed coat mucilage structure [14]. We examined whether mutations in *IAR4* could suppress these additional *fei1 fei2* phenotypes. In contrast to its role in the root, *iar4* did not suppress the increased hypocotyl width phenotype of *fei1 fei2* (Fig. 5A and 5B). In fact, the *iar4-5* mutant also had slightly wider hypocotyls and this effect was additive with that of *fei1 fei2*. The additive nature of *iar4-5* and *fei1 fei2* on hypocotyl width suggests that these genes may act in parallel to regulate cell wall function. Unlike in the hypocotyl, mutations in *iar4-5* did not affect the seed coat mucilage of *fei1 fei2*. The seed coat mucilage of the triple *fei1 fei2 iar4-5* mutant resembled that of *fei1 fei2* indicating that mutations in *IAR4* do not suppress this phenotype (Fig. 6).

**Figure 7:**
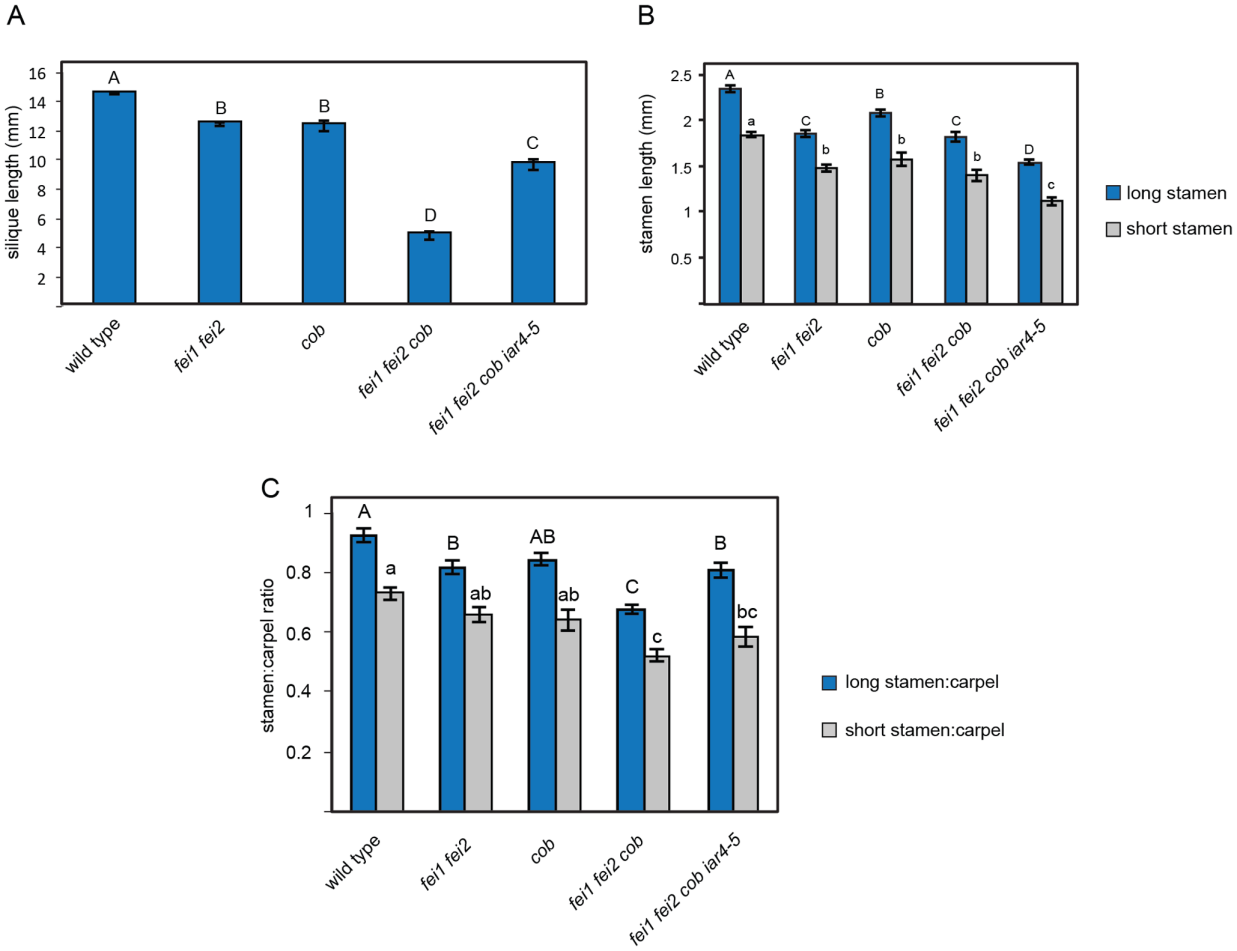
Effect of *iar4-5* on floral phenotypes of *fei1 fei2*. (**A**) Silique length from indicated genotypes. Plants were grown on soil under 16h light regime for four weeks at 22°C. The first five fully developed siliques from the primary inflorescences were used for the analysis. Values are the mean ± SE (n = 25). (**B**) Stamen length and (**C**) Carpel:stamen ratio of indicated genotypes. Stamens and carpels were removed from individual flowers and measured. Values are the mean ± SE (n>11). Different letters indicate significant differences between groups. Data were analyzed with one-way ANOVA and Tukey's post-hoc comparisons; *P*<0.05.

An additional role for the FEI RLKs is to act additively with COBRA (COB) in stamen filament elongation in the flower. *COBRA* encodes a GPI-anchored protein that associates with the cell wall and is required for the oriented deposition of cellulose in rapidly expanding cells [22]. Like *fei1 fei2, cob-1* mutants are deficient in cellulose and as a result display a short, swollen root phenotype that is enhanced by elevated sucrose. Although neither the *fei1 fei2* nor *cob-1* mutants themselves display an obvious floral phenotype, a triple *fei1 fei2 cob-1* mutant has short stamen filaments and as a result is partially infertile [12]. Similar to root cells in the elongation zone, cells of the stamen filament also undergo primarily longitudinal expansion. Therefore, we assessed the ability of *iar4-5* to suppress the short stamen phenotype of *fei1 fei2 cob-1* mutant. Analysis of a quadruple *fei1 fei2 cob-1 iar4-5* mutant indicated that the *iar4-5* allele restores fertility in *fei1 fei2 cob-1* (Fig. 7A; S4 in File S1). We hypothesized that the restoration of fertility is a result of increased stamen filament length in the quadruple mutant. However, analysis of flower parts showed that in the *fei1 fei2 cob-1 iar4-5* stamen length is further reduced (Fig. 7B), indicating that *iar4-5* does not in fact restore stamen filament length to the *fei1 fei2 cob-1* mutant. The ratio of carpel length to stamen length is increased in the quadruple mutant (Fig. 7C) relative to the infertile *fei1 fei2 cob-1 iar4-5* parent, suggesting that the fertility is most likely rescued due to further decrease in carpel length in *fei1 fei2 cob-1 iar4-5* flowers (Fig. S4 in File S1) rather than increased stamen length.

### *iar4* is a general suppressor of defects in cell wall synthesis

**Figure 8:**
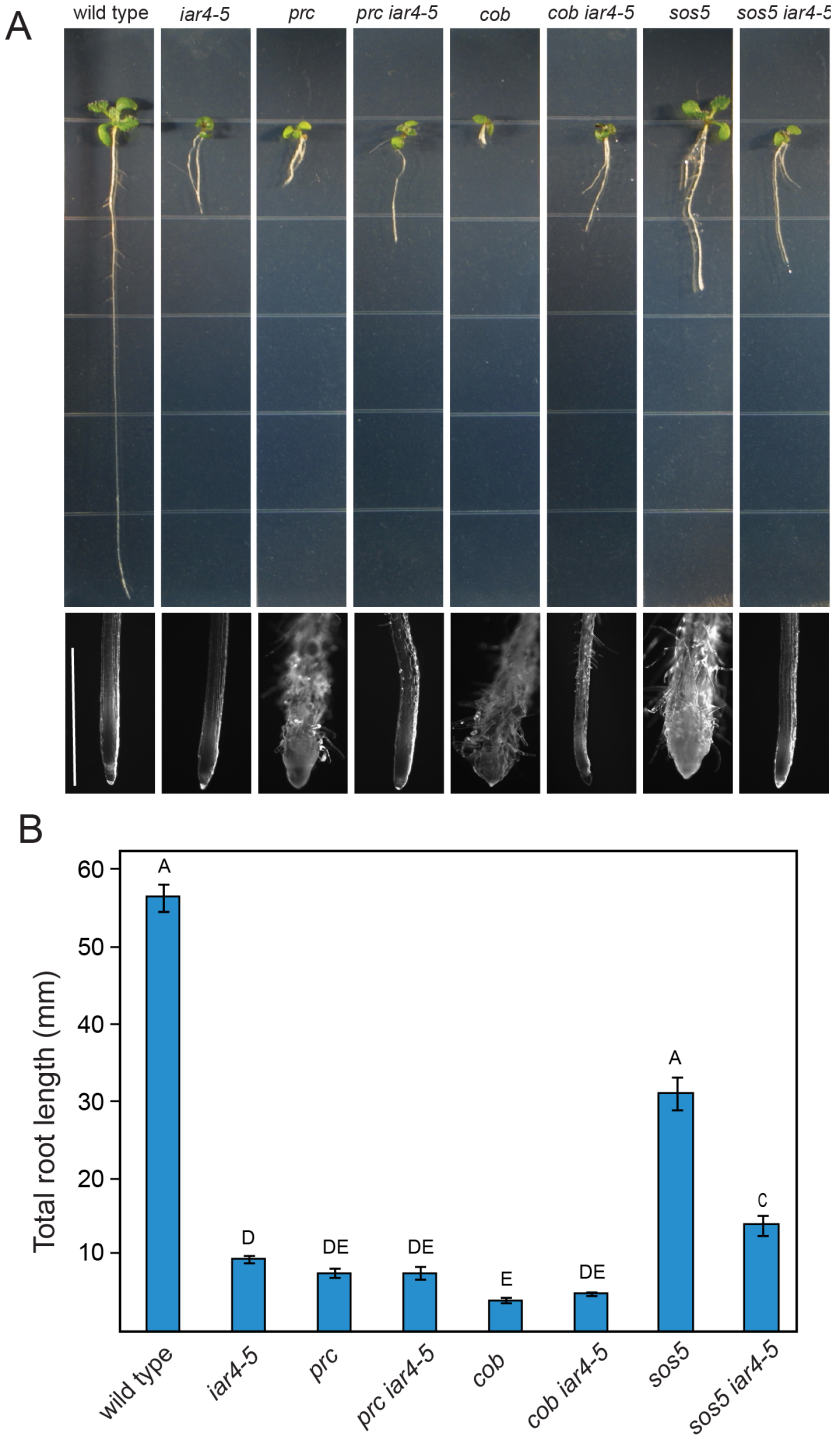
Mutations in *iar4-5* suppress other cell wall mutants. (**A**) Phenotypes of indicated seedlings grown for 14 days on MS medium supplemented with 4.5% sucrose. The bottom is a close-up of the root tips of the seedlings shown above. Scale bar = 1.5 mm (**B**) Quantification of total root elongation. Plants were grown on MS medium supplemented with 4.5% sucrose for 10 days and total root lengths were measured. Values represent means ± SE (n>17). Different letters indicate significant differences between groups. Data were analyzed with one-way ANOVA and Tukey's post-hoc comparisons; *P*<0.05.

To ascertain whether loss-of-function mutations in *IAR4* suppress defects in cell expansion exhibited by other cell wall mutants or whether they are specific to the FEI pathway, we crossed *iar4-5* to *sos5, procuste (prc*; a null allele of *CESA6*), and a weak allele of *cobra*, (*cob-1*). When grown in the presence of 4.5% sucrose, each of these mutants displayed a substantial reduction in root length accompanied by radial expansion of cells in the root tip as a result of reduced cellulose biosynthesis. As expected, *iar4* suppressed swelling of the cells in the root tip of *sos5*, which acts in the FEI pathway (Fig. 8). However, in contrast to the *fei1 fei2 iar4* triple mutant, which displayed a substantial suppression of the root elongation defect observed in both parental lines, the *sos5 iar4* double mutant displays even shorter roots than *sos5* single mutant. Thus, the root of the *sos5 iar4* double mutant is short, but not swollen and thus resembles the *iar4* parental root phenotype. *iar4-5* also suppresses the swollen root phenotypes of both the *cob-1* and *prc* mutants, both of which affect cellulose synthesis independent of the FEI pathway (Fig. 8A), and in both cases it moderately restores the root elongation in these mutants (Fig. 8B).

**Figure 9:**
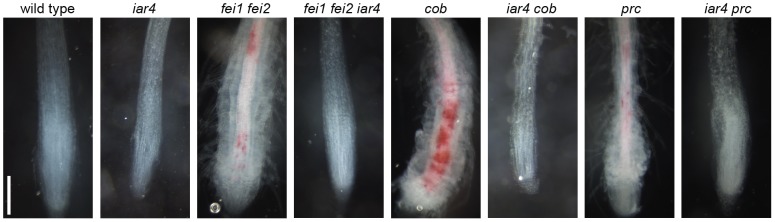
*iar4* suppresses lignin accumulation in cell wall mutants. Phloroglucinol stain for lignin (red) accumulation in root tips of seedlings of the indicated genotype grown on 4.5% sucrose for 2 weeks. Scale bar = 1 mm.

We next tested whether mutations in *IAR4* could suppress the accumulation of ectopic lignin in these mutants. Lignin is deposited ectopically into the cell wall in response to decreased cellulose synthesis that occurs in cellulose deficient mutants. Previous studies have shown that the roots of *fei1 fei2, cob-1*, and *prc* all accumulate ectopic lignin [12], [20], [23], [24]. Interestingly, when we assessed the roots of these cell wall mutants in an *iar4-5* background using a colorimetric stain, no ectopic lignin deposition was observed (Fig. 9). This result is consistent with the suppression of the root swelling defect in these mutants by *iar4* and suggests that IAR4 is required for the ectopic deposition of lignin that occurs in response to decreased cellulose biosynthesis. Taken together, these observations suggest that *iar4* is not specific to the FEI pathway, but rather acts as a more general suppressor of defects in cellulose biosynthesis.

## Discussion

We demonstrate that reducing auxin function, either through loss-of-function mutations in *IAR4* or in the auxin biosynthetic genes *WEI8* and *TAR2*, suppresses the root swelling that occurs in the *fei1 fei2* mutant. Several lines of evidence suggest that *iar4*, and by inference, auxin, acts not in the FEI pathway directly, but rather independently to regulate cell wall function. First, *iar4* acts additively with *fei1 fei2* to increase hypocotyl width and affect floral development. Second, *iar4* reverts the swollen root phenotype and suppresses the accumulation of ectopic lignin in other cellulose synthesis mutants such as *cob-1* and *prc*, which act in parallel with the FEIs. Finally, the *iar4* mutation confers resistance to isoxaben, which inhibits cellulose synthase function rather than the FEI pathway. The data support a model in which reduced auxin function acts to modulate cell wall function in the root in some way to counteract the effects of reduced cellulose synthesis in the *fei1 fei2* mutant root, as well in other cellulose deficient mutants.

The two aspects of cell expansion are its orientation and extent. The amount and orientation of cellulose microfibrils in the cell wall determines to a large extent the orientation of expansion. In roots, cellulose microfibrils are orientated transversely around the cells, restricting radial expansion leading to principally longitudinal cell expansion [25]. Reduction of cellulose levels, in mutants such as *fei1 fei2* or *prc*, results in a loss of the restriction of radial expansion, and therefore swollen roots. Less is known regarding the regulation of extent of cell expansion. The driving force for cell expansion is the turgor pressure of the cell, which provides the force for increasing the volume of the cell that is opposed by the rigidity of cell wall, which is controlled by the breaking and reforming of the bonds holding the polymers of the cell wall together. Mutants have been isolated that are defective in root elongation that affect the orientation of cell expansion, the extent of cell expansion, or both processes [26].

Previous studies have linked auxin to the regulation of cell wall function in aerial tissues primarily by affecting the extent of cell expansion. The acid-growth hypothesis attributes auxin-induced cell expansion to the acidification of the cell wall, which results in an increase in the activity of the wall loosening enzymes expansins [27]. Expansins disrupt the non-covalent bonds that form between cellulose and hemicelluloses in the wall and thus promote cell expansion in hypocotyls and modulate the growth of leaves, petioles, and roots [27]–[29]. Auxin Binding Protein (ABP1) may play an important role in this response; ABP1 activates H^+^ ATPases and K^+^ channels at the plasma membrane upon the perception of auxin and is required for cell elongation [30]. In addition to expansins, other wall-loosening enzymes such as xyloglucan hydrolases (XGH) and endotransglycosylases (XET), which cleave and re-graft a major form of hemicellulose, xyloglucan, are also activated upon acidification of the cell wall and in response to auxin [31]. Consistent with these findings, the mechanical extensibility of epidermal cells isolated from azuki bean epicotyls increases dramatically following incubation with XGH [32]. These are among many studies that support a role for auxin in increasing the extensibility of the cell wall in the shoot.

In contrast to the shoot, auxin appears to inhibit root growth and exogenous auxin causes a rapid alkalization of the apoplast in the elongation zone of Arabidopsis roots [33], [34]. However, the auxin-insensitive, gain-of-function Aux/IAA mutant, *axr3-1* has a shorter root as compared to the wild type, similar to the short root phenotype of the *iar4* single mutants. Further, auxin represses numerous genes involved in cell wall synthesis and remodeling. Among the genes that are de-regulated in *axr3-1* seedlings treated with IAA are those that encode arabinogalactan proteins (AGPs), expansins (EXP), extensins, proline rich proteins (PRP), xyloglucan endotransglucosylase-hydrolases (XTHs), and pectin methyl-esterases (PMEs) [35]. Although extensins rigidify the cell wall, a disproportionate number of genes repressed in *axr3-1* encoded proteins that loosen the cell wall matrix and thus promote cell elongation [36]. Similarly, mutations in the auxin influx carrier, *lax3*, prevent the induction of expansin expression in the in developing lateral roots of Arabidopsis seedlings. *LAX3* is required for lateral root initiation and its expression precedes the necessary changes in cell wall architecture that are predicted to play a critical role in the emergence of lateral root primordium [37]. The lack of wall plasticity coupled with alterations in the expression of genes that encode cell wall remodeling proteins in the *axr3-1* and *lax3* mutants suggest that auxin may also promote wall loosening in roots. Recently, a detailed study of gene expression kinetics in response to auxin in Arabidopsis roots revealed that a large number of genes involved in cell wall remodeling are regulated in response to exogenous auxin application [38]. Notably, one cluster of auxin down-regulated genes included 57 genes annotated as playing a role in cell wall synthesis/remodeling, including CESA2, suggesting that auxin modulates cell wall synthesis in roots.

The growth of seedlings in the presence of auxin has been shown to lead to root swelling in a manner independent of ethylene biosynthesis [39], [40]. This suggests that exogenous auxin decreases the integrity of the cell wall, leading to a loss of growth anisotropy. This is consistent with the suppression of swelling in mutants defective in cellulose biosynthesis by reduction of endogenous auxin that is described here. An important question is by what mechanism does exogenous auxin increase root swelling in wild-type roots, and conversely, how does reduced endogenous auxin suppress swelling in cellulose-deficient roots. One possibility is that auxin negatively regulates cellulose synthesis. Consistent with this, the transcript level of the *CESA2* gene has recently been shown to be decreased in response to auxin [38]. However, the suppression of the *procuste* mutant by *iar4* in our study makes this somewhat unlikely as *procuste* is a null allele of CESA6. However, it is possible that reduced auxin levels may elevate cellulose synthesis via alternative CESA complexes as CESA6 acts redundantly with CESA2, CESA5, and CESA9 in some Arabidopsis tissues [41]. This scenario is unlikely, at least with respect to CESA5 because mutations in *IAR4* do not suppress the defects in seed coat mucilage production in *fei1 fei2* where CESA5 is required for cellulose biosynthesis in the seed coat [14]. A somewhat more plausible model is that in the long term, auxin modulates the rigidity of the wall not by regulating cellulose synthesis, but by altering other properties of the wall, such as the crosslinking of cellulose microfibrils or the activity of extensins or other cell wall modifying enzymes as described above. Intriguingly, while both *iar4* and *fei1 fei2* mutant roots are short in the presence of elevated sucrose, the triple *iar4 fei1 fei2* mutant displays both a non-swollen root, as well as root elongation comparable to the wild type. In contrast, although mutations in *iar4* suppress the swollen root phenotypes of the other cell wall mutants, they do not fully restore root elongation in *prc, cob,* or *sos5*. This suggests that *iar4* does not simply restore cellulose biosynthesis in these mutants, as this would rescue both the swollen root and root length phenotypes. Additionally, it is conceivable the short root phenotype of *iar4* may be partially attributed to decreased cell division in the root apical meristem as auxin has been shown to regulate both the activity and the size of the meristem in the Arabidopsis root [42]. While we isolated *iar4* as a suppressor of *fei1 fei2*, the short root phenotype of both *fei1 fei2* and the *iar4* mutant but not the *fei1 fei2 iar4* triple mutant raises an interesting question. How does *fei1 fei2* suppress *iar4*? It is unlikely that it does so by suppressing any potential effects of *iar4* on cell division rates, and thus likely does so through modulation of cell wall properties.

**Figure 10:**
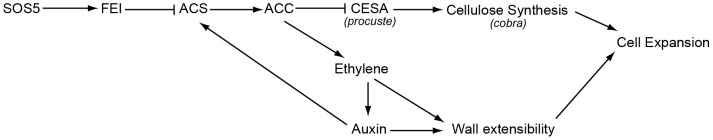
Model of the FEI pathway. Hypothetical model depicting the role of both auxin and the FEI pathway in regulating cell expansion. See text for additional details.

We propose a model that combines the previously characterized role of the FEI receptor-like kinases in regulating cellulose synthesis with a role for auxin in regulating cell wall rigidification in the root (Fig. 10). We have previously shown that ACC may act as a signal in the FEI pathway to regulate cellulose biosynthesis. The *fei1 fei2* mutations lead to radial cell expansion in the root as a result of decreased cellulose synthesis, which alters wall function such that there is not sufficient force to constrict radial expansion. One model consistent with the data is that decreased auxin results in an increase in the rigidity of the cell wall, which allows the reduced levels of cellulose in the *fei1 fei2* mutant to provide sufficient force to inhibit radial expansion. In most cases this increase in rigidity would also cause a decrease in the overall expansion of the cells, and hence a decrease in the length of the root, such as in observed in the *cob-1 iar4* and *prc iar4* lines. We hypothesize that in the case of the *fei1 fei2 iar4* line, the increased rigidity caused by decreased auxin is precisely balanced by the reduced cellulose levels cause by the *fei1 fei2* mutations, leading to both a lack of swelling and near wild-type elongation. Auxin increases the expression of multiple *ACS* genes [43], which may also have an effect on the FEI signaling pathway. Alternatively, auxin could be involved in the signaling cascade linking perception of perturbation of the cell wall to changes in cell wall synthesis. Interestingly, a recent study has demonstrated that the inhibition of root cell elongation that occurs in response to isoxaben is attenuated by mutations in the *tir1-1* auxin receptor and growth in the presence of the synthetic antagonist of TIR1, PEO-IAA. Furthermore, results from this study indicated that inhibitors of the precursor to ethylene, ACC, fully restore growth anisotropy in the presence of isoxaben and this effect was shown to act independent of ethylene [44]. Consistent with this data, inhibitors of ACC, but not ethylene suppress the swollen root phenotype of *fei1 fei2* [12].

While the data suggest a role of ACC independent of ethylene in regulating cell wall function, ethylene itself clearly also plays a role in cell expansion. Growth of Arabidopsis seedling in the presence of ethylene causes a reduction in cell expansion in many tissues, including the root. In pea epicotyls, ethylene causes a reorientation of the deposition of the cellulose microfibrils in the cortical cells from primarily transverse to primarily longitudinally [45]. In Arabidopsis, ethylene shifts the orientation of cell expansion in the hypocotyl of etiolated seedlings, but not the extent of cell expansion the hypocotyls from etiolated seedlings grown in the presence of ethylene are substantially shorter, which is balanced by an increase in hypocotyl width. In contrast, growth of etiolated seedlings in ethylene causes a strong reduction in root elongation that is likely the results of effects on both the extent and orientation of expansion as the reduction in root elongation is only partly matched by an increase in root width.

The characterization of *iar4* in this study as a suppressor of defects in cell wall synthesis provides evidence that auxin plays a key role in the regulation of primary cell wall function and suggests that wall extensibility may be a major determinant of cell expansion in the root. Whether auxin acts as a general regulator of cell wall function throughout development or participates in the active signaling processes that occur in response to perturbations in the cell wall remains an interesting question for future studies.

## Materials and Methods

### Plant Material and Growth Conditions

All lines used in this study are in the Columbia (Col-O) ecotype of *Arabidopsis thaliana*, except where noted. The *shou2-3* (SALK_091909) allele was obtained from the SALK T-DNA insertional collection [46]. The *prc-1* [24] and *cob-1* [47] mutants were obtained from the Arabidopsis Biological Resource Center. The *wei8* and *tar2-1* mutants have been previously described (Stepanova *et al*., 2008). For *in vitro* studies, seeds were surface sterilized, cold treated for 4 days at 4°C, germinated on vertical plates containing 1 x Murashige and Skoog (MS) salts, 0.6% phytagel (Sigma, St Louis, MO, USA) and either 0% or 4.5% sucrose and grown at 22°C under constant light. For the analysis of root elongation, seeds were germinated on 4.5% sucrose and total root elongation was quantified using ImageJ [48]. For the analysis of auxin sensitivity, seedlings were grown for 4 days on MS media, then transferred to new MS medium supplemented with various auxin levels and root elongation quantified four days after transfer. For the hypocotyl elongation assay, seedlings were exposed to light for 3 hours and grown for 4 days in the dark on MS agar supplemented with 1% sucrose. The width of each hypocotyl was measured 1 mm from the hook of an etiolated seedling. For growth on soil, plants were grown either under constant light or long day conditions at 23°C. For growth in the presence of isoxaben, seedlings were germinated and grown in the absence of sucrose for 5 days then transferred to MS agar supplemented with 0 nM (DMSO control), 1 nM or 2 nM isoxaben for 48 hours. The root width was measured at the level of youngest root hair using ImageJ [48].

### Positional Cloning of *shou2*

The *fei1* and *fei2* mutations (Columbia, Col ecotype) were introgressed into Landsberg erecta (L*er*) through back crossing with L*er* six times and a line homozygous for *fei1* and *fei2* was obtained. Theoretically, after six backcrosses, approximately 98.4% of the genome is L*er*, with the exception of regions around the *fei1* and *fei2* mutations, which remain Col. We tested 42 molecular markers across all 5 chromosomes and found only the molecular markers F6NI8 and TI0P12 (close to *FEI1*), and TIJ8 (close to *FEI2*) remained Col. All other 39 markers were homozygous for the L*er* SNPs. A mapping population was generated by crossing *fei1 fei2 shou2-1* (Col) to *fei1 fei2* (L*er*). Bulk segregant analysis was performed using a total of 42 markers that span the Arabidopsis genome on a pool of DNA obtained from 40 F_2_ seedlings showing suppression of the *fei1fei2* phenotype. The mutation was initially mapped to an interval spanning markers FI2K8 (7.954Mbp) and FI3K9 (9.744Mbp) on chromosome 1. Fine mapping was facilitated by the root hair phenotype of *shou2* mutants using restriction fragment length polymorphisms and cleaved amplified polymorphic sequence markers. The *shou2-1* mutation was mapped to a ∼47-kilobase (kb) region delimited by recombination events between marker F3I6-D (8.552 Mbp) and F3I6-F (8.599Mbp) of chromosome 1. Sequencing of 12 genes within this region identified mutations in the first and seventh exons of At1g24180 in *fei1 fei2 shou2-1* and *fei1 fei2 shou2-2* respectively.

### Phloroglucinol Staining

Seedlings were fixed in a solution of three parts ethanol: one part acetic acid for fifteen minutes and transferred to 70% ethanol for 10 minutes. Seedlings were then cleared in chlorohydrate:glycerol:water (8∶1∶2) for 5 minutes and stained for a total of 5 minutes in a 2% phloroglucinol-HCl solution.

### Microscopy and seed staining

The calcofluor stain was done as described by Willats *et al*. [49]. Seeds were pre-treated with 50 mM EDTA, stained for 20 min in 25 µg/ml fluorescent brightener 28 (Sigma), washed overnight in water and then visualized using a Zeiss LSM710 confocal microscope equipped with a 405 nm laser diode. Pontamine staining was done as described by Anderson *et al*. [50]. Seeds were stained for 30 min in 0.01% Pontamine fast scarlet S4B (Sigma) following a 90 min pre-hydration, washed for 4 hours in water and then visualized using a Zeiss LSM710 confocal microscope equipped with a 561 laser. Flowers and root tips were imaged using bright field microscopy and hypocotyls using dark field microscopy on the compound Leica microscope. Cross sections of the root elongation zone were prepared and imaged as described by Xu *et al*. [12].

### Flower and silique analyses

Plants were grown for 4 weeks under the long day conditions. For the silique measurements we used first five siliques from primary inflorescences. For the analysis of flower parts we used stamens and carpels dissected from fully matured flowers. Siliques, stamen filaments and carpel lengths were measured using ImageJ software [48]. The significance of the data was tested by one-way ANOVA with Tukey's post-hoc comparison.

### Gene expression analysis

100 mg of root tissue from 8d-old seedlings were harvested and snap frozen in liquid nitrogen. RNA was isolated using the RNeasy extraction kit (Qiagen) and genomic DNA was removed using on-column DNase digestion (Qiagen). 1 µg total RNA was used for cDNA synthesis conducted with random hexamers using the iScript cDNA synthesis kit (Bio-rad). Real-time reverse transcription PCR was performed using the Applied Biosystems ViiA RT-PCR system, the SYBR *Premix Ex Taq* (Takara) and *IAR4* (AT1G24180) specific primers. Relative mRNA values were calculated using the 2^-ΔΔCt^ method [51] with *TUB4* (AT5G44340) as an internal reference gene. Primer sequences are listed in Table S2 in File S1.

## Supporting Information

File S1 **Supplemental material.** Figure S1, T-DNA insertion in *IAR4* results in reduced transcript levels. (A) Cartoon of the *IAR4* gene: boxes represent exons, line introns. The site of the T-DNA insertion in *iar4-7* is indicated by the red triangle and primers used for the analysis of transcript levels indicated below. (B) *IAR4* transcript levels of different regions (as indicated in (A)); n = 3 (± SE). Transcript levels were calculated using the 2^(−ΔΔCt)^ method as described [51]. Asterisks indicate significant differences between *iar4-7* and the wild type or *fei1 fei2 iar4-7* and *fei1 fei2* (*P*<0.05); nd, not detectable. Figure S2, Complementation test of *iar4* alleles. F_1_ progeny from indicated crossed were grown on MS media containing 4.5% sucrose for 14 days. Note that the roots of the F_1_ seedlings display a non-swollen (i.e. suppressed) phenotype, suggesting that the *iar4-5, iar4-6 and iar4-7* mutations are allelic. Scale bar = 0.5 mm. Figure S3, Effect of altered auxin levels on *fei1 fei2* root growth and on the suppression of *fei1 fei2* by *iar4*. (A) Effect of elevated temperature on the suppression of root swelling of the *fei1 fei2* mutant by *iar4-5*. Four-day old seedlings grown on MS with no sucrose at 22°C were transferred to MS media containing 4.5% sucrose and grown an additional five days at 28°C. (B) Quantification of root elongation of wild-type and *fei1fei2* in response to auxin. Four-day-old seedlings were transferred to media containing the indicated level of auxin and the amount the roots grew after transfer was measured four days later. Values represent the mean of ± SE (n>15). (C) Quantification of relative root elongation of wild-type and *fei1fei2* as in described in A. Values were normalized to the no auxin control. Data were analyzed by Student's t-test; *, *P*<0.05. **, *P*<0.01. The experiment was repeated three times and showed very similar results. Figure S4, *iar4-5* restores fertility of *fei1 fei2 cob-1*. (A) Inflorescence, (B) flower and (C) silique phenotypes of indicated genotypes. Note that *iar4-5* partially restores the silique length of *fei1 fei2 cob*. Plants were grown on soil under long day conditions for four weeks. To visualize stamen length, some petals and sepals were removed from each flower. Bar = 1 mm. (D) Carpel length of indicated genotypes. Different letters indicate significant differences between groups. Data were tested with one-way ANOVA and Tukey's post-hoc analysis; n>13; *P*<0.05. Table S1, Markers used to map *iar4-5*. Table S2, Primers used for gene expression analysis.
(PDF)
