# Correction: Alterations in Auxin Homeostasis Suppress Defects in Cell Wall Function

**DOI:** 10.1371/journal.pone.0110992

**Published:** 2014-10-08

**Authors:** The PLOS ONE Staff

Figure 8 is incorrect. The authors have provided a corrected version here.

**Figure 8:**
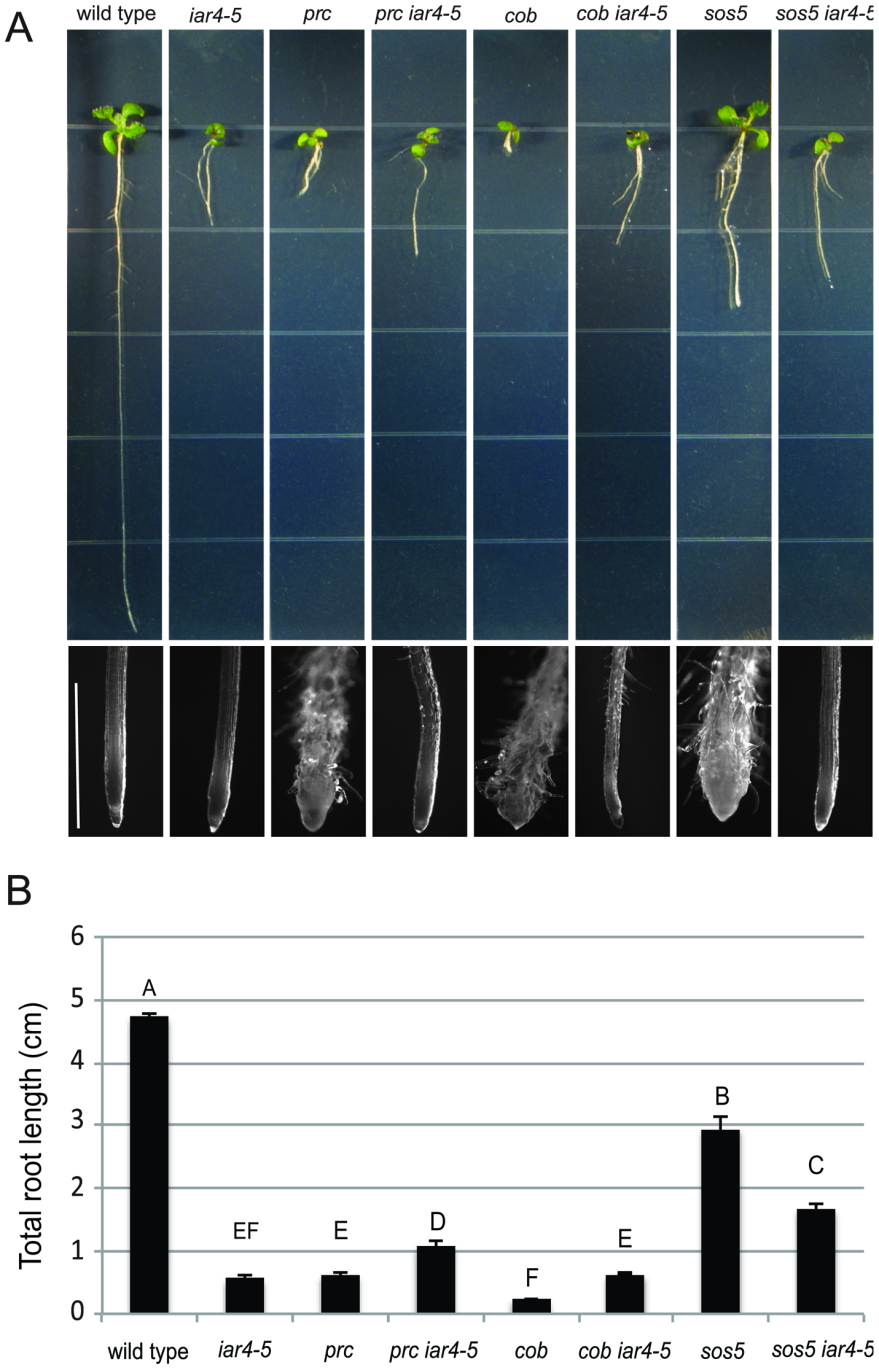
Mutations in iar4-5 suppress other cell wall mutants. (A) Phenotypes of indicated seedlings grown for 14 days on MS medium supplemented with 4.5% sucrose. The bottom is a close-up of the root tips of the seedlings shown above. Scale bar  =  1.5 mm (B) Quantification of total root elongation. Plants were grown on MS medium supplemented with 4.5% sucrose for 10 days and total root lengths were measured. Values represent means ± SE (n>17). Different letters indicate significant differences between groups. Data were analyzed with one-way ANOVA and Tukey's post-hoc comparisons; P<0.05.
