# Supplementary material for: Alterations in Auxin Homeostasis Suppress Defects in Cell Wall Function
Source: PLoS One. 2014 May 23;9(5):e98193. doi: 10.1371/journal.pone.0098193 (PMC4032291; doi:10.1371/journal.pone.0098193)
Supplement: File S1 — Supplemental material. Figure S1, T-DNA insertion in IAR4 results in reduced transcript levels. (A) Cartoon of the IAR4 gene: boxes represent exons, line introns. The site of the T-DNA insertion in iar4-7 is indicated by the red triangle and primers used for the analysis of transcript levels indicated below. (B) IAR4 transcript levels of different regions (as indicated in (A)); n = 3 (± SE). Transcript levels were calculated using the 2(−ΔΔCt) method as described [51]. Asterisks indicate significant differences between iar4-7 and the wild type or fei1 fei2 iar4-7 and fei1 fei2 (P<0.05); nd, not detectable. Figure S2, Complementation test of iar4 alleles. F1 progeny from indicated crossed were grown on MS media containing 4.5% sucrose for 14 days. Note that the roots of the F1 seedlings display a non-swollen (i.e. suppressed) phenotype, suggesting that the iar4-5, iar4-6 and iar4-7 mutations are allelic. Scale bar = 0.5 mm. Figure S3, Effect of altered auxin levels on fei1 fei2 root growth and on the suppression of fei1 fei2 by iar4. (A) Effect of elevated temperature on the suppression of root swelling of the fei1 fei2 mutant by iar4-5. Four-day old seedlings grown on MS with no sucrose at 22°C were transferred to MS media containing 4.5% sucrose and grown an additional five days at 28°C. (B) Quantification of root elongation of wild-type and fei1fei2 in response to auxin. Four-day-old seedlings were transferred to media containing the indicated level of auxin and the amount the roots grew after transfer was measured four days later. Values represent the mean of ± SE (n>15). (C) Quantification of relative root elongation of wild-type and fei1fei2 as in described in A. Values were normalized to the no auxin control. Data were analyzed by Student's t-test; *, P<0.05. **, P<0.01. The experiment was repeated three times and showed very similar results. Figure S4, iar4-5 restores fertility of fei1 fei2 cob-1. (A) Inflorescence, (B) flower and (C) silique phenotypes of in [file pone.0098193.s001.pdf]

Table S1: Markers used to map *shou2*

Marker[1]	Position	Enzyme	Size of predicted product (bps) Col	Ler	Oligonucleotide primers
F5O8	8.392	*XbaI*	145 + 251	396	CCAGTTGTTCAGGAAATGGAA<br>TGACGAATGTATTGCAACCG
T23E23D	8.442	*Bsp12861*	537	346 + 191	GTGATCTTGCGCCAGAAGTA<br>CAACCTGATTGTCTGCCTCA
F3I6-E	8.53	*NdeI*	320	134 + 186	CCGAACCAACCTTGAATTTG<br>TTGGTGTGCCGATAAAAACA
F3I6-D	8.552	*MseI*	215 + 35 +23	250 + 22	TGCCATGTCGTAAATTCCTG<br>GCAGAATAAGCCATCGTGGT
F3I6H	8.57	*BsrDI*	173	153	TTCAGTTCACGATTAAAATTGCAAT<br>TCTTCTCAGCTGTTTCGTCG
F316F	8.599	*BsrDI*	524	195 + 329	GGGACCTCGTTACCCAAAAT<br>GCTTCAACACTCCTCCAAATC
F316-C	8.607	-	197	182	TTGTCGAAGGGACAGTGTTG<br>GTGGTCTGCTCTCAGCCTCT

[1]Note: all markers are on chromosome 1.

Table S2. Primers used for gene expression study.

**Gene**	**Primer name**	**Sequence 5’→3’**
IAR4	102	TGGCGTTATCACGACTTTCTT
IAR4	103	GCGTGACGGTGATTCACATA
IAR4	104	TCGGAGGAGATCTTAGCCTTT
IAR4	105	TTTTGGTAATCGCCGCTTC
IAR4	130	TCGCCCTCTCCTAACTCTTTC
IAR4	131	GTAATCGCCGGCTTTAGGA
TUB4	TUB4F	ACCAATGAAAGTAGACGCCA
TUB4	TUB4R	AGAGGTTGACGAGGAGATGA

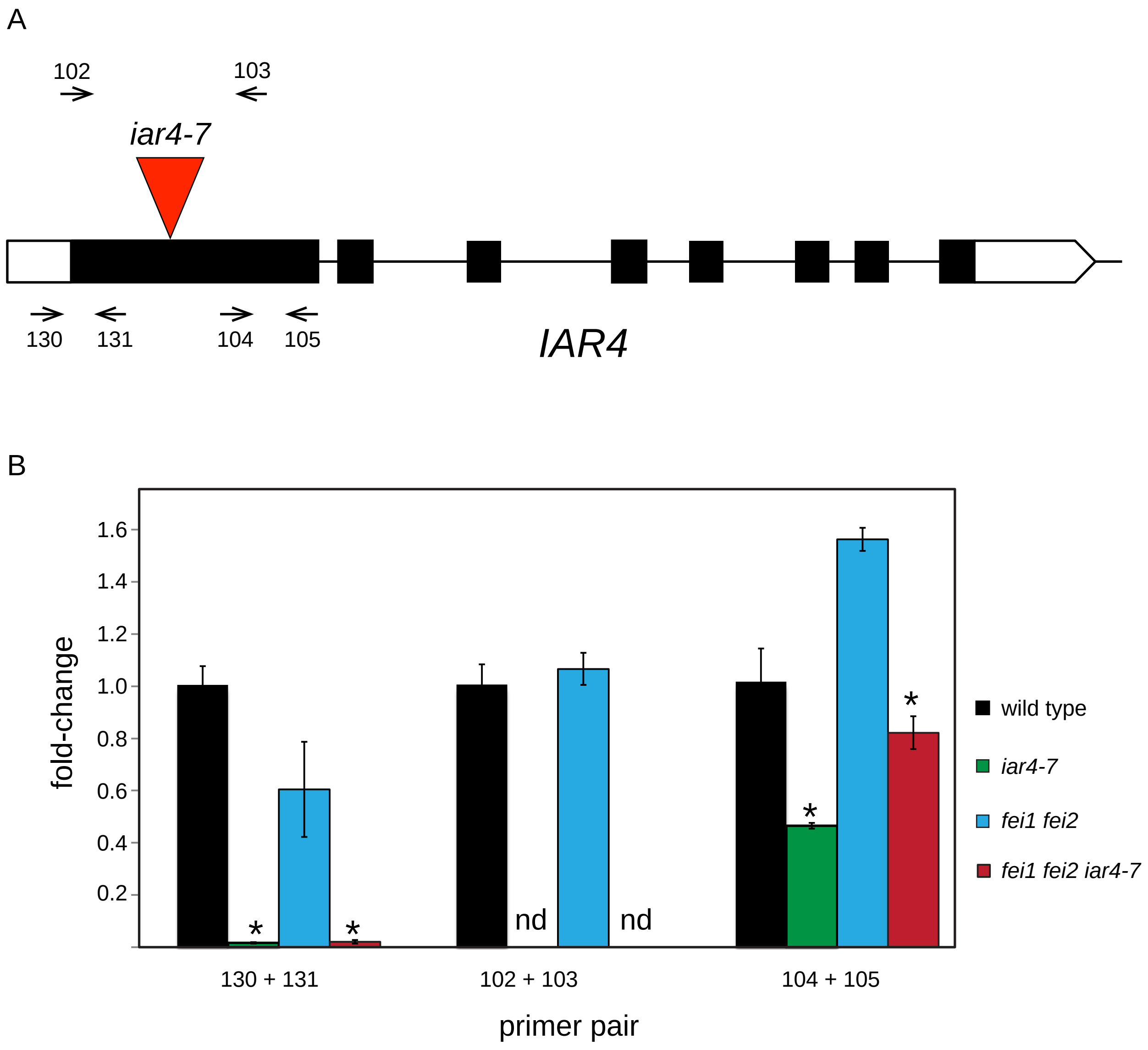

A
102
103
iar4-7
130
131
104
105
IAR4
B
fold-change
1.6
1.4
1.2
1.0
0.8
0.6
0.4
0.2
*
*
nd
nd
*
*
130 + 131
102 + 103
104 + 105
primer pair
wild type
iar4-7
fei1 fei2
fei1 fei2 iar4-7

Figure S1

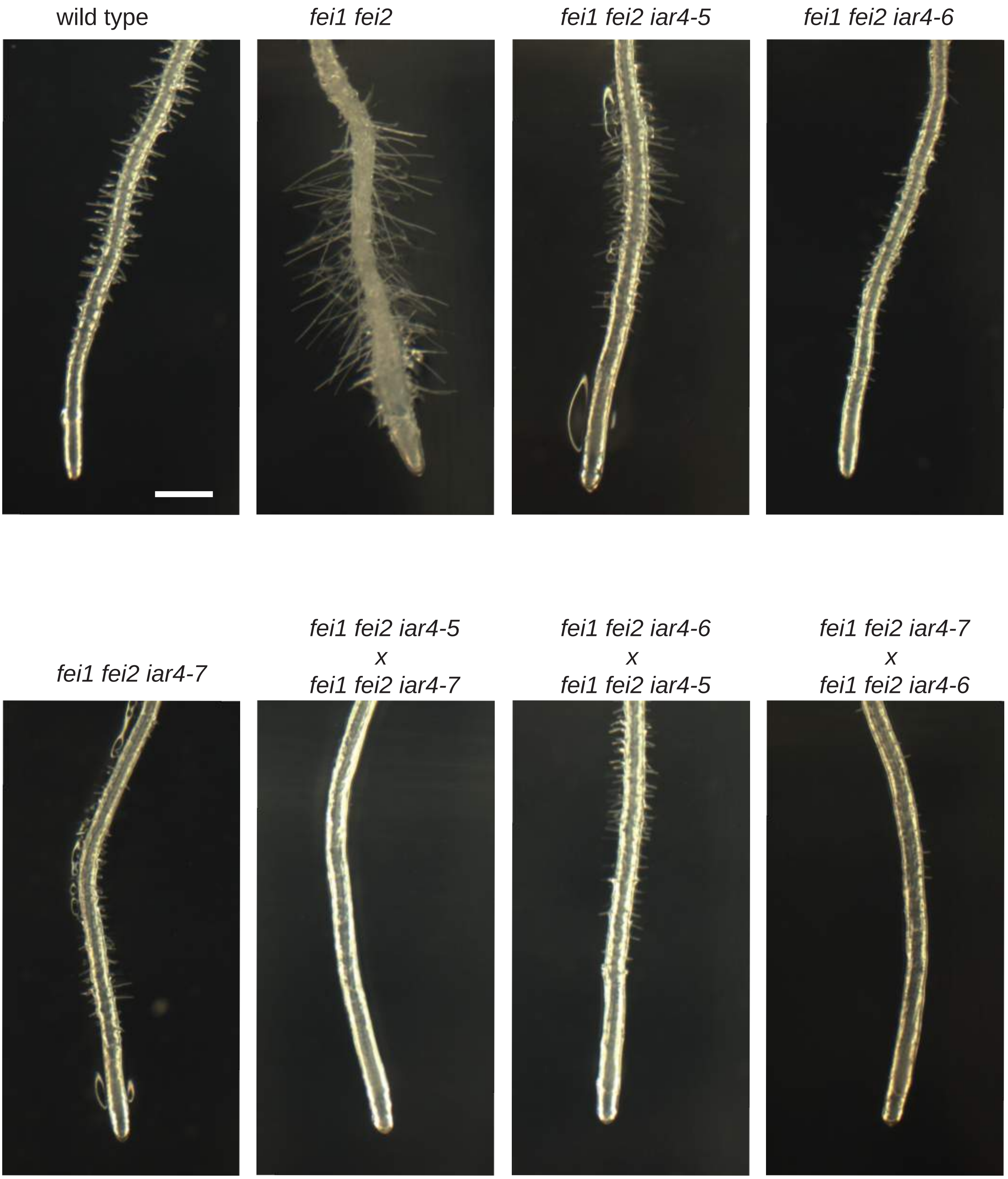
wild type
fei1 fei2
fei1 fei2 iar4-5
fei1 fei2 iar4-6
fei1 fei2 iar4-7
fei1 fei2 iar4-5 x fei1 fei2 iar4-7
fei1 fei2 iar4-6 x fei1 fei2 iar4-5
fei1 fei2 iar4-7 x fei1 fei2 iar4-6

Figure S2

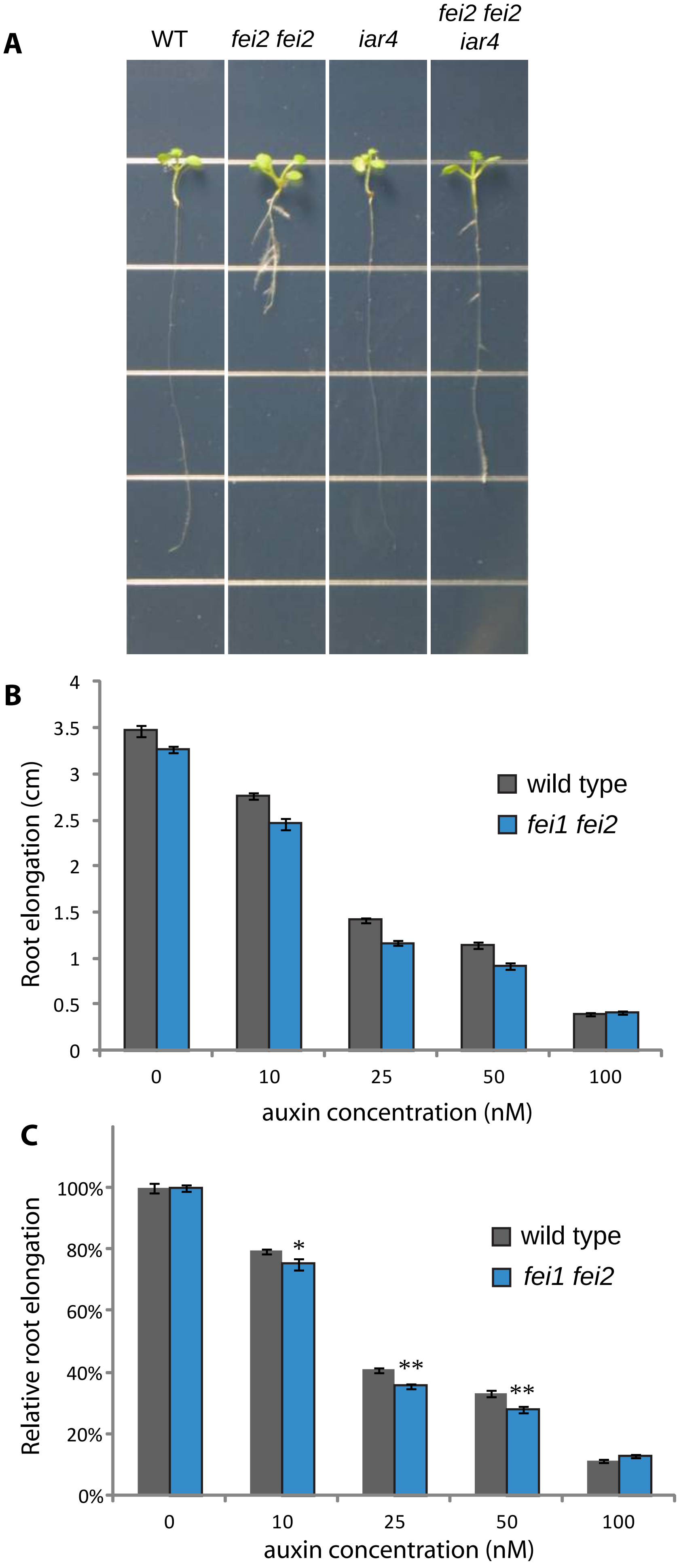
A
WT
fei2 fei2
iar4
fei2 fei2 iar4
B
Root elongation (cm)
4
3.5
3
2.5
2
1.5
1
0.5
0
wild type
fei1 fei2
0
10
25
50
100
auxin concentration (nM)
C
Relative root elongation
100%
80%
60%
40%
20%
0%
*
**
**
wild type
fei1 fei2
0
10
25
50
100
auxin concentration (nM)

Figure S3

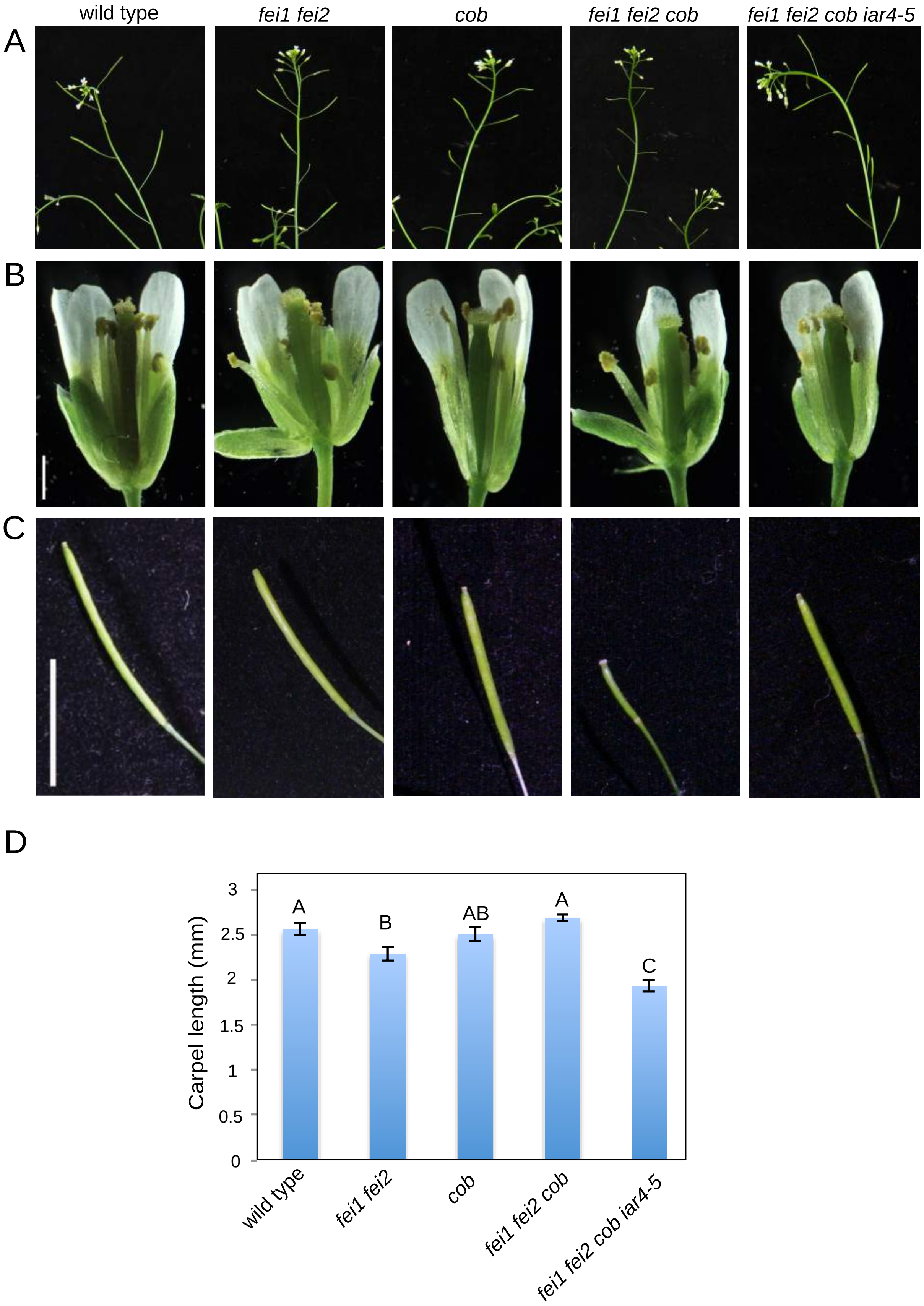
wild type
fei1 fei2
cob
fei1 fei2 cob
fei1 fei2 cob iar4-5
A
B
C
D
Carpel length (mm)
3
2.5
2
1.5
1
0.5
0
A
B
AB
A
C
wild type
fei1 fei2
cob
fei1 fei2 cob
fei1 fei2 cob iar4-5

Figure S4
